# A newly noninvasive model for prediction of non-alcoholic fatty liver disease: utility of serum prolactin levels

**DOI:** 10.1186/s12876-019-1120-z

**Published:** 2019-11-27

**Authors:** Pengzi Zhang, Wenghuan Feng, Xuehui Chu, Xitai Sun, Dalong Zhu, Yan Bi

**Affiliations:** 1 Department of Endocrinology, Drum Tower Hospital Affiliated to Nanjing University Medical School, No 321, Zhongshan Road, Nanjing, 210008 Jiangsu China; 2 Department of General Surgery, Drum Tower Hospital Affiliated to Nanjing University Medical School, Nanjing, China

**Keywords:** Nonalcoholic fatty liver disease, Prolactin, Diagnostic model

## Abstract

**Backgrounds:**

To investigate the value of prolactin (PRL) in diagnosing non-alcoholic fatty liver disease (NAFLD).

**Methods:**

Metabolic parameters and serum PRL levels were measured in 452 males and 421 females, who were randomized to the estimation or the validation group as a 1:1 ratio. Hepatic steatosis was diagnosed via abdominal ultrasound. Variables that significantly associated with NAFLD in univariate analysis were included in multiple logistic regression. We used the receiver operator characteristic (ROC) curves to test the model performance. Besides, 147 patients underwent metabolic and liver biopsy were analyzed to validate the diagnostic value of this model.

**Results:**

Body mass index, alanine aminotransferase, prolactin, high density lipoprotein cholesterol and HbA1c were included into models. In males, the area under ROC curve (AUC) was 0.86 (95%CI: 0.82–0.91) for the validation group. With two cut-off points (− 0.79 and 1.71), the sensitivity and specificity for predicting NALFD was 95.2 and 91.1% in the validation group, respectively. In females, the AUC was 0.82 (95%CI: 0.76–0.88) for the validation group. With two cut-off points (− 0.68 and 2.16), the sensitivity and specificity for predicting NALFD was 97.1 and 91.4% in the validation group, respectively. In subjects with liver pathology, the AUC was higher than that of fatty liver index. A positive correlation between the scores of the model and the severities of NAFLD was observed. Importantly, we demonstrated a potential value of this model in predicting nonalcoholic steatohepatitis.

**Conclusion:**

We established a mathematic model that can conveniently and effectively diagnose the existence and severities of NAFLD.

## Background

Non-alcoholic fatty liver disease (NAFLD) refers to fat accumulation more than 5% of hepatocyte, in the absence of excess alcohol intake, virus hepatitis and drug induced liver injury, including simple steatosis, nonalcoholic steatohepatitis (NASH), fibrosis and, ultimately, cirrhosis [1]. It is estimated that the global prevalence of NAFLD was 25.2%, and NAFLD was associated with a series of metabolic comorbidities [2]. Early diagnosis of NAFLD and NASH is of great significance, since advanced stage of NAFLD and NASH had a higher carotid artery intima–media and thickness and overall mortality [1, 3]. Liver histology is regarded as the most reliable method of detecting NAFLD, however, the risk of biopsy-related complications including severe pain, peri-procedural hypotension and bleeding limit its use in clinical practice [4]. Although there are non-invasive techniques for assessing hepatic steatosis (ultrasound, ^1^H-magnetic resonance spectroscopy, and computed tomography), these procedures were time-consuming and costly and therefore often unavailable for screening NALFD in large population-based studies [5]. Subsequent studies have proposed several indexes such as fatty liver index (FLI) for diagnosing NAFLD [6], yet the calculation of these markers were complicated. Prolactin (PRL) is a pituitary-derived hormone which was recently shown to be closely associated with the existence and progression of fatty liver [7]. Here we aimed to develop a method to predict the presence of NAFLD based on data from subjects enrolled in two separate cohort studies and evaluated whether the involvement of PRL would improve the diagnostic value than previous reported indexes.

## Subjects and methods

### Subjects information

In the first cohort study, a total of 452 male and 421 female subjects aged between 18 and 80-year-old and received abdominal ultrasound examination in our department from September 2015 to February 2017 were enrolled. In the second cohort study, 147 eligible patients who received bariatric surgery from March 2017 to August 2017 were analyzed. The exclusion criteria were excessive alcohol consumption (≥210 g/week in men and ≥ 140 g/week in women), virus hepatitis (detected by specific markers of hepatitis or self-reported virus hepatitis history), autoimmune hepatitis, type 1 diabetes, history of steatogenic (e.g. tetracycline), antipsychotic and contraceptive medication, hyperthyroidism or hypothyroidism; pregnancy and lactation; hyperprolactinemia; systematic corticosteroids therapy; malignant tumors; sever hepatic or renal dysfunction; and pituitary diseases. The study project was approved by Nanjing Drum Tower Hospital Committee on research with human subjects and written informed consent was obtained from each participant. This study was also registered on ClinicalTrials.gov (NCT03296605).

### Clinical and biochemical measurements

Anthropometric measurements including body mass index (BMI), waist circumference, and blood pressure were recorded for each individual, and fasting blood sample (8–10 h overnight) was collected for laboratory analysis. Fasting plasma glucose (FBG) and HbA1c were measured via a hexokinase method (TBA-200FR, Tokyo, Japan) and high-performance liquid chromatography (HLC-73G8, Tosoh, Japan), respectively. Alanine aminotransferase (ALT), aspartate transaminase (AST), triglycerides (TG), total cholesterol (TC), low-density lipoprotein (LDL) cholesterol, and high-density lipoprotein (HDL) cholesterol were measured through an autoanalyser (Abbott Laboratories, Parsippany, USA). PRL was measured on an automated chemiluminescent immunoassay (Siemens Immulite 2000, UK) using fasting blood samples drawn in the morning. The coefficients of intra- and inter-assay variation were between 2.49–3.47% and 2.91–3.14%, respectively. The laboratory’s standard reference intervals of PRL are < 25μg/l for women and < 20μg/l for men. In the first cohort study, all subjects received abdominal ultrasonography (Philips HD15 Ultrasound Unit, Amsterdam, Netherlands) by the experienced sonographer from our hospital who were blinded to the patients’ information. The diagnosis of steatosis is based on the following ultrasonographic patterns: “bright liver”; increased echo contrast between hepatic and renal parenchyma; vessel blurring or poor visualization of diaphragm [8].

Menopausal status was defined as follows: pre-menopausal: all females less than 40 years of age and between 40 and 60 years who reported menstrual cycle; post-menopausal: all females over 60 years and between 40 and 60 years without self-reported menstrual cycle [9].

The calculation of FLI was according to previous literature [10], which includes four variates: BMI, TG, γ-glutamyl-transferase (GCT), waist circumference (WC).

$$ \frac{{\mathrm{e}}^{0.953\ast \ln\ \left(\mathrm{TG}\right)+0.139\ast \mathrm{BMI}+0.718\ast \ln\ \left(\mathrm{GGT}\right)+0.053\ast \mathrm{WC}-15.745}}{1+{\mathrm{e}}^{0.953\ast \ln\ \left(\mathrm{TG}\right)+0.139\ast \mathrm{BMI}+0.718\ast \ln\ \left(\mathrm{GGT}\right)+0.053\ast \mathrm{WC}-15.745}}\ast 100 $$

### Liver pathology examination

In the second cohort study, liver samples from each patient were obtained during the surgery and prepared for haematoxylin and eosin staining. The liver histology was assessed by two liver pathologists who were blinded to the patients’ clinical data, and hepatic steatosis was defined as the proportion of affected hepatocytes is ≥5%. Histopathologic percentage of steatosis was graded as mild (5–33%), moderate (33–66%), or severe (> 66%). NAFLD activity score (NAS) includes steatosis (0–3), lobular inflammation (0–3), and ballooning (0–2). NASH was diagnosed when NAS ≥ 5, and was excluded if NAS < 3 [11].

### Statistical analysis

Because of gender differences in PRL levels and NAFLD incidence, demographic and laboratory data for males and females were analyzed separately [12]. The protocol of this study is in accordance with the standards for reporting diagnostic accuracy (STARD) statement [13]. The sample size was estimated based on the following parameters [14]: α = 0.05, marginal error = 0.1, the prevalence of NAFLD is around 25%, pre-determined values of sensitivity and specificity is around 80%.

Data were presented as median with interquartile range (IQR). The Mann-Whitney U and Kruskal Wallis test were used to compare non-normal distribution data between two or multiple groups, respectively.

For the formulation of predictive models, univariate logistic regression analysis was carried out on variables of patients with or without NAFLD in the estimation group. Variables significantly associated with the presence of NAFLD in univariate analysis (*P* < 0.05) were then subjected to multivariate logistic analysis to identify factors independently associated with NAFLD. Both forward selection and backward elimination were utilized in stepwise regression analysis. The predictive model was built by modeling the values of the independent factors and their regression coefficient. Finally, using variables except PRL, we also established another regression model for predicting NAFLD. Diagnostic efficiency between these models were performed by calculating the integrated discrimination improvement (IDI) and categorical net reclassification improvement (NRI) [15] in subjects with liver biopsy.

The receiver operating characteristic (ROC) curve was used to assess the diagnostic value of the model. ROC curve is the plot of all the 1-specificity (horizontal axis) versus its sensitivity (vertical axis). Each dot on the curve represents a possible cut-point at a decision threshold [16]. The optimal cut-off points were chosen from the ROC curve and we selected two cut-off points to achieve sensitivity over 90% or specificity over 90%. The final model was then applied to the validation set to test the accuracy.

Statistical analysis of the data were executed in SPSS software (Version 22.0; SPSS Inc., USA). By virtue of a nonparametric Delong test, which employed the theory developed for generalized U-statistics [17], the comparison between our model and FLI was accomplished in MedCalc (Version 12.7, MedCalc Ostend Belgium). IDI and NRI analysis were conducted in R version 3.5.1 (R Foundation for Statistical Computing, Vienna, Austria). *P* value < 0.05 was considered significant.

## Results

### Subjects characteristics

The flow chart of subjects screening was shown in Additional file 1: Figure S1. In the first cohort study, a total of 452 males and 421 females were included (Additional file 1: Figure S1). Compared with non-NAFLD group, significantly higher levels of BMI, SBP, waist, HbA1c, FBG, ALT, AST, TG, TC, and lower levels of HDL and PRL were observed in NAFLD patients (Additional file 4: Table S1).

After randomization, 226 males (54.4% were NAFLD) and 210 females (52.4% were NAFLD) were chosen into the estimation group, and 226 males (55.3% were NAFLD) and 211 females (50.2% were NAFLD) were selected into the validation group. There were no significant differences among almost all variates between the estimation group and the validation group in both genders (Table 1).

**Table 1:** Clinical and laboratory data of the estimation and validation groups

	Men			Women		
	Estimation group	Validation group	*P*	Estimation group	Validation group	*P*
N	226	226		210	211	
Age (years)	54 (44, 62)	53 (41.5, 61.5)	0.25	56 (46, 64.3)	55 (45, 64)	0.33
BMI (kg/m^2^)	25.1 (22.9, 27.2)	25.6 (23.4, 27.5)	0.99	134 (120, 150)	134 (121, 146)	0.77
SBP (mmHg)	131 (119, 144)	137 (127, 149.5)	0.01	80 (70, 91.3)	80.5 (71, 89)	0.68
DBP (mmHg)	80 (72, 88)	82 (76, 91)	0.18	24.2 (21.9, 27.6)	24.8 (22.3, 27.5)	0.32
Waist (cm)	94 (88, 98.3)	95 (88, 99)	0.99	89 (80, 98)	88.5 (82.3, 96)	0.85
HbA1c (%)	8.1 (6.5, 9.9)	7.1 (5.9, 9.4)	0.15	7.6 (5.6, 9.4)	6.8 (5.4, 8.7)	0.06
FBG (mmol/L)	7.4 (5.6, 9.2)	6.8 (5.3, 8.8)	0.41	6.1 (4.8, 8.4)	5.6 (4.7, 7.8)	0.10
ALT (U/L)	25.3 (16.9, 41.7)	23.1 (16.6, 34.7)	0.47	20.2 (14.5, 32.4)	19 (13.9, 27.8)	0.21
AST (U/L)	19.7 (16.1, 25.2)	18.3 (15.3, 23.1)	0.33	19.3 (16, 24.6)	18.4 (15, 23.7)	0.15
TG (mmol/L)	1.5 (1.1, 2.4)	1.5 (1.1, 2.2)	0.86	1.4 (1, 2)	1.4 (1, 2)	0.66
TC (mmol/L)	4.4 (3.6, 5.1)	4.5 (3.7, 5)	0.68	4.5 (3.8, 5.3)	4.5 (3.8, 5.2)	0.99
HDL (mmol/l)	1 (0.8, 1.2)	1 (0.8, 1.2)	0.99	1.1 (1, 1.4)	1.1 (1, 1.4)	0.64
LDL (mmol/l)	2.4 (1.8, 3)	2.5 (1.9, 3)	0.45	2.5 (1.9, 3.2)	2.4 (2, 3.1)	0.84
PRL (ug/L)	8.8 (6.5, 12.1)	7.9 (6.3, 10.5)	0.28	9.7 (6.7, 14.2)	9.9 (6.6, 14)	0.98

*BMI* Body mass index, *SBP* Systolic blood pressure, *DBP* Diastolic blood pressure, *FBG* Fasting blood glucose, *HbA1c* Haemoglobin 1c, *ALT* Alanine aminotransferase, *AST* Aspartate transaminase (AST), *HDL* High-density lipoprotein, *LDL* Low-density lipoprotein, *PRL* Prolactin, *TC* Total cholesterol, *TG* triglyceride. Data are shown as median with interquartile range (IQR). *p* values are based on Mann-Whitney U test

### Estimation of the model in diagnosing NAFLD

**Fig. 1:**
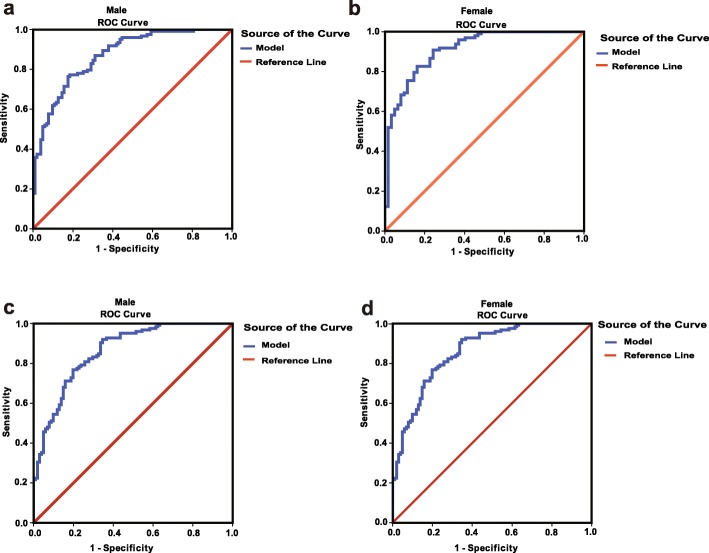
ROC curves of NAFLD in different subgroups. **a** ROC curve in the estimation group of males, the AUC is 0.87 (95%CI: 0.83–0.92) (*n* = 226). **b** ROC curve in the estimation group of females, the AUC is 0.91 (95%CI: 0.87–0.96) (*n* = 210). **c** ROC curve in the validation group of males, the AUC is 0.86 (95%CI: 0.82–0.91) (*n* = 226). **d** ROC curve in the validation group of females, the AUC is 0.82 (95%CI: 0.76–0.88) (*n* = 211)

In the estimation group of males, DBP, BMI, waist, FBG, HbA1c, PRL, ALT, AST, TG and HDL were identified as predictors of NAFLD by univariate analysis (Table 2). Then all these variables were included in a forward multivariate logistic regression, and factors that independently associated with NAFLD were identified: BMI, PRL, ALT and HDL (Table 2). We constructed a formula combining these four variables as follows: 0.474*BMI (kg/m^2^) - 0.131*PRL (μg/l) + 0.026*ALT (U/l) -2.139*HDL (mmol/l) - 8.758. The area under ROC (AUC) was 0.87 (95%CI: 0.83–0.92) (Fig. 1a). Two cut-off points were selected to rule out (<− 0.79) and rule in (> 1.71) NAFLD. Applying a lower cut-off of − 0.79 would exclude NAFLD with a high accuracy (sensitivity and negative predictive values (NPV) of 95.9 and 91.9%, respectively). On the other hand, using a high cut-off of 1.71 would predict NAFLD with high accuracy, with specificity and positive predictive values (PPV) of 95.1 and 92.6%, respectively. In this cohort, 91.9% (57/62) of subjects with a score < − 0.79 were correctly classified as not having NAFLD, while 92.6% (63/68) of participants were correctly classified as having NAFLD with a score > 1.71 (Table 3).

**Table 2:** Binary logistic regression analysis in the estimation group

	Male				Female			
	Univariate analysis		Multivariate analysis		Univariate analysis		Multivariate analysis	
	Odds ratio (95%CI)	*P*	Odds ratio (95%CI)	*P*	Odds ratio (95%CI)	*P*	Odds ratio (95%CI)	*P*
Age	0.987 (0.968, 1.006)	0.182	–	> 0.05	1.027 (1.007, 1.047)	0.008	–	> 0.05
SBP	1.009 (0.995, 1.023)	0.212	–	> 0.05	1.012 (0.999, 1.025)	0.082	–	> 0.05
DBP	1.029 (1.007, 1.051)	0.008	–	> 0.05	1.006 (0.987, 1.025)	0.531	–	> 0.05
BMI	1.524 (1.336, 1.740)	< 0.001	1.606 (1.329, 1.942)	< 0.001	1.392 (1.245, 1.556)	< 0.001	1.471 (1.198, 1.808)	< 0.001
Waist	1.161 (1.101, 1.225)	< 0.001	–	> 0.05	1.119 (1.075, 1.164)	< 0.001	–	> 0.05
PRL	0.882 (0.822, 0.945)	< 0.001	0.877 (0.791, 0.973)	0.013	0.828 (0.774, 0.886)	< 0.001	0.786 (0.678, 0.912)	0.001
FBG	1.264 (1.122, 1.423)	< 0.001	–	> 0.05	1.499 (1.292, 1.740)	< 0.001	–	> 0.05
HbA1c	1.149 (1.019, 1.296)	0.024	–	> 0.05	1.634 (1.369, 1.951)	< 0.001	1.682 (1.261, 2.244)	< 0.001
ALT	1.022 (1.009, 1.036)	0.001	1.027 (1.009, 1.045)	0.004	1.051 (1.026, 1.078)	< 0.001	1.062 (1.011, 1.116)	0.016
AST	1.029 (1.003, 1.056)	0.027	–	> 0.05	1.083 (1.035, 1.133)	0.001	–	> 0.05
TG	2.141 (1.526, 3.004)	< 0.001	–	> 0.05	1.810 (1.244, 2.633)	0.002	–	> 0.05
TC	1.099 (0.854, 1.415)	0.464	–	> 0.05	1.075 (0.826, 1.399)	0.591	–	> 0.05
HDL	0.052 (0.017, 0.160)	< 0.001	0.118 (0.027, 0.512)	0.004	0.177 (0.068, 0.464)	< 0.001	–	> 0.05
LDL	1.112 (0.800, 1.545)	0.527	–	> 0.05	1.240 (0.894, 1.720)	0.198	–	> 0.05

*BMI* Body mass index, *SBP* Systolic blood pressure, *DBP* Diastolic blood pressure, *FBG* Fasting blood glucose, *HbA1c* Haemoglobin 1c, *ALT* Alanine aminotransferase, *AST* Aspartate transaminase (AST), *HDL* High-density lipoprotein, *LDL* Low-density lipoprotein, *PRL* Prolactin, *TC* Total cholesterol, *TG* Triglyceride

**Table 3:** The sensitivity and specificity of the model in the estimation and validation group

	Cut-off	Estimation group					Validation group				
		non NAFLD	NAFLD	Sensitivity (%)	Specificity (%)	AUC (95%CI)	non NAFLD	NAFLD	Sensitivity (%)	Specificity (%)	AUC (95%CI)
Male	> − 0.79	46	118	95.9	55.3	0.87 (0.83–0.92)	46	119	95.2	54.5	0.86 (0.82–0.91)
	<−0.79	57	5				55	6			
	> 1.71	5	63	51.2	95.1		9	64	51.2	91.1	
	< 1.71	98	60				92	61			
Female	> − 0.68	26	104	94.5	74	0.91 (0.87–0.96)	41	102	97.1	61.3	0.82 (0.76–0.88)
	<−0.68	74	6				65	3			
	> 2.16	3	57	51.8	97		5	52	34.0	91.4	
	< 2.16	97	53				53	101			

*NAFLD* Non-alcoholic fatty liver disease, *AUC* Area under the ROC curve

In the estimation group of female subjects, we found that age, BMI, waist, FBG, HbA1c, PRL, ALT, AST, TG and HDL were significantly associated with NAFLD (Table 2). Further multivariate analysis showed that BMI, PRL, HbA1c, FBG, and ALT were independently associated with NAFLD (Table 2). We constructed a formula combining these four variables as follows: 0.386*BMI (kg/m^2^) - 0.24*PRL (μg/l) + 0.52*HbA1c (%) + 0.06*ALT (U/l) - 11.619. The AUC was 0.91 (95%CI: 0.87–0.96) (Fig. 1b). Two cut-off points were selected to diagnose the presence (> 2.16) and absence (<− 0.68) of NAFLD. Applying the lower cut-off (− 0.68), 104 (94.5%) of 110 patients with NAFLD were correctly identified. The presence of NAFLD could be excluded with high certainty since only 6 of the 80 (7.5%) with a score below − 0.68 had NAFLD based on ultrasound (NPV = 92.5%). In the estimation group, there were 60 subjects with a score higher than 2.16. By using the high cut-off (2.16), 97 (97%) of 100 non-NAFLD subjects were correctly excluded, and 57 of 60 subjects (PPV = 95%) with NAFLD were correctly diagnosed (Table 3). A total of 78 males and 70 females with a score between their respective two cut-off points were considered indeterminate. Based on our score, hepatic ultrasound would have been obviated in 65% (148/226) of the males and 67% (140/210) of the females.

In addition, we also tested backward regression analysis and both forward selection and backward elimination gave the same regression model when predicting the presence of NAFLD (data not shown), suggesting that variates included in our model were stable.

### Validation of the NALFD diagnosis model

To validate the accuracy of this model, we first applied the two models into the validation groups. The AUC in male and female validation group is 0.86 (95%CI: 0.82–0.91) and 0.82 (95%CI: 0.76–0.88), respectively (Fig. 1c, d). In males, the lower cut-off point of − 0.79 predicted NAFLD with a sensitivity of 95.2% and specificity of 54.5%. For the high cut-off point of 1.71, the sensitivity and specificity are 51.2 and 91.1%, respectively (Table 3). In females, the lower cut-off point of − 0.68 gave a sensitivity of 97.1% and specificity of 61.3%. For the high cut-off point of 2.16, the sensitivity and specificity for diagnosing NAFLD is 34.0 and 91.4%, respectively (Table 3). In the validation group, hepatic ultrasound would have been obviated in 59% (134/226) of the males and 59% (125/211) of the females.

Since there is evidence that PRL concentrations were affected by age in males [12] and by menopausal status in females [18], we also tested our model in men younger or older than 50 years older and in women with pre- or post-menopause separately. The clinical and laboratory data are listed in Additional file 5: Table S2. Elder men displayed significantly higher levels of age, SBP, HbA1c and HDL while lower DBP, ALT and TG levels than younger males. Levels of age, SBP, FBG, HbA1c, TG, TC and LDL in postmenopausal women were significantly higher while DBP and PRL levels were significantly lower than premenopausal women. The AUC (95%CI) of our model in males younger than 50 years old was 0.88 (0.85–0.92) (Additional file 2: Figure S2a) and 0.82 (0.75–0.89) (Additional file 2: Figure S2b) in those who were equal or greater than 50. The AUC (95%CI) of our model in pre- and postmenopausal women were 0.93 (0.88–0.97) (Additional file 2: Figure S2c) and 0.79 (0.74–0.85) (Additional file 2: Figure S2d), respectively (all *P* < 0.01).

### Validate in subjects with liver biopsy

In the second cohort study, 49 male and 98 female patients who received bariatric surgery and met the inclusion and exclusion criteria were subjected to liver biopsy. According to the histology examination, 7 males and 13 females were non-NAFLD (Additional file 3: Figure S3).

**Fig. 2:**
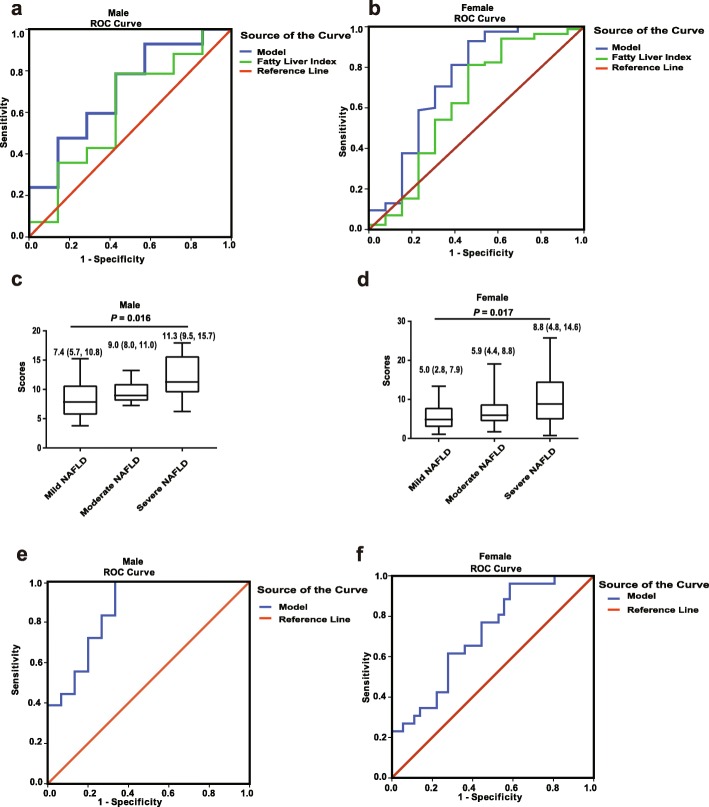
Performance of established models in subjects received liver biopsy. **a** and **b** Comparison of ROC curves between our model and fatty liver index in males and females, respectively. **c** and **d** The scores in male and females categorized into different severities of NAFLD, *P* values are based on Kruskal-Wallis test. **e** and **f** ROC curve of our model in identifying subjects with NASH in males and females, respectively

In males, significantly higher levels of FBG, ALT, AST and NAS scores were more common in NAFLD patients (Additional file 6: Table S3). Female NAFLD patients exhibited higher levels of FBG, HbA1c, ALT and NAS scores and lowers levels of PRL. Using liver pathology as gold standard benchmark, we tested the model for defining cases of NAFLD and found that the AUC remained high in males (0.71, 95%CI: 0.56–0.83) and females (0.74, 95%CI: 0.56–0.92) (Fig. 2a, b). Next, we compared the diagnostic efficiency between this model and FLI, and the results showed that the AUC of our model was higher than that of FLI (males: 0.62 (95%*CI*, 0.47–0.75, z statistic = 2.249, *P* = 0.02; females: 0.63, 95%*CI*: 0.44–0.83, z statistic = 2.118, *P* = 0.03) (Table 4).

**Table 4:** Comparison of the performance of current formula and FLI

	Male			Female		
	AUC (95%CI)	*z* statistic	*P* value	AUC (95%CI)	z statistic	*P* value
Current model	0.71 (0.56–0.83)	2.249	0.02	0.74 (0.56–0.92)	2.118	0.03
FLI	0.63 (0.47–0.75)			0.63 (0.44–0.83)		

*FLI* Fatty liver index, *AUC* Area under the ROC curve

Further, subjects were categorized into three groups based on the proportion of hepatic steatosis. We found that higher score levels were associated with a more severe NAFLD. (Fig. 2c, d).

Importantly, 18 males and 26 females had a NAS score ≥ 5 were diagnosed as NASH (Additional file 7: Table S4), and we investigated whether our model had a potential value in predicting NASH. We found that the AUC in identifying NASH is 0.86 (95%CI 0.74–0.99) in males and 0.71 (95%CI 0.59–0.84) (Fig. 2e, f). Two cut-off points were selected to achieve sensitivity of 94.4% (cut-off 7.42, specificity 66.7%) or specificity of 93.3% (cut-off 12.06, sensitivity 33.9%) for predicting NASH in males. For females, the two cut-off points were 3.67 (sensitivity of 96.2% and specificity of 41.3%) and 10.49 (sensitivity of 30.8% and specificity of 90%).

### Comparison of regression models with and without PRL

We further determined the incremental predictive value of adding PRL in our model for identifying subjects with hepatic steatosis. Thus, we established another formula using variables without PRL (age, BMI, SBP, DBP, waist, HbA1c, FBG, ALT, AST, TG, TC, HDL, LDL) in both genders. By means of forward stepwise logistic analysis, the formulas without PRL were constructed: 0.469*BMI (kg/m2) + 0.028*ALT (U/l) -2.236*HDL (mmol/l)-9.838 for males and 0.264*BMI (kg/m2) + 0.072*waist (cm) + 0.481*HbA1c (%) + 0.084*ALT (U/l)-17.467 for females (Additional file 8: Table S5). To compare the performance of the formula with and without PRL, we calculated categorical net reclassification improvement (NRI) and the integrated discrimination improvement (IDI) in subjects with liver biopsy. These parameters are considered as new standards for deciding incremental capacity of predictors [19]. Remarkable improvements in discrimination were confirmed by the IDI (0.196; 95%CI, 0.052–0.340; *P* = 0.008 for males and 0.262; 95%CI, 0.090–0.434; *P* = 0.003 for females), suggesting further average separation of NAFLD from non NAFLD by adding PRL. The addition of PRL also led to a net reclassification of NAFLD patients in the appropriate directions, shown by an increase in category NRI. NRI in males was not significant (0.119; 95%CI, − 0.361-0.599; *P* = 0.627), but there was a statistical improvement in reclassification for females (0.500; 95%CI, 0.187–0.813; *P* = 0.002 for females) (Additional file 8: Table S5).

## Discussion

In this study, we attempted to develop an approach for diagnosing NAFLD via common clinical and laboratory data. We have demonstrated that by using the following equation: 0.474*BMI (kg/m^2^) - 0.131 * PRL (μg/l) + 0.026*ALT (U/l) -2.139*HDL (mmol/l) - 8.758, and two cut-off points (− 0.79 and 1.71) in males, and 0.386*BMI (kg/m^2^) - 0.24 * PRL (μg/l) + 0.52*HbA1c (%) + 0.06*ALT (U/l) - 11.619, and two cut-off points (− 0.68 and 2.16) in females, NAFLD can be identified with a high sensitivity and specificity both in the estimation group and validation group. The ORs of PRL in the model of males and females is 0.877 and 0.786 (Table 2), indicating that an increase of 1 SD in PRL was associated with a reduced risk of 12.3% in males and 21.4% in females for NAFLD when the other variables in the model were kept constant.

NAFLD is a chronic liver disease that may lead to fibrosis and cirrhosis if without early intervention [20]. Our study included not only 873 well-characterized individuals in whom hepatic steatosis was identified through abdominal ultrasound, but also 147 patients who have received liver biopsy, the gold standard for diagnosing NAFLD and NASH. Our models consisted following parameters: BMI, HbA1c, PRL, ALT and HDL, in which BMI, HbA1c were shown to be risk factors for NAFLD, while HDL was shown to be negatively associated with NAFLD [21–23]. ALT levels reflect the inflammation state of liver, and subjects with elevated liver enzymes (ALT) are recommended to be evaluated for presence of NASH [24, 25]. Moreover, an inverse association between PRL and NAFLD was observed in our model. PRL is a polypeptide hormone mainly produced from anterior pituitary, well known for its lactogenic properties [26]. Recent studies also suggested an important role of PRL in metabolic disease, and PRL was proven to be a protective factor against the existence and progress of NAFLD [27, 28], which were supported by our current data. PRL is a hormone that has diurnal variation and varies through the menstrual cycle. To exclude this variability, fasting serum samples were collected in all subjects on 8:00 to minimize the influence of environmental stress. In addition, we applied our model in both pre- and postmenopausal females and found that the AUC were higher than 0.7 in both groups.

Previous evidence described that serum PRL levels were beginning to decline with the growth of age. In males, a study carried out in middle-aged men suggested that PRL concentration was negatively correlated with age [29]. However, another study demonstrated that changes of PRL in males after 50 years old did not reach statistical difference [12]. Since aging is also a risk factor for NAFLD [30], we then inspected the influence of aging on PRL. Here we divided male subjects into two groups according to whether their ages were more than 50 years old (Additional file 8: Table S5). We found that there were no significant difference in PRL concentrations between two groups. Moreover, age did not enter the final model in multivariate logistic regression analysis in both genders, suggesting that after adjusting for other confounding factors, age was not independently associated with NAFLD. We also tested the diagnostic efficiency of our models in these subgroups and the AUC were higher in both younger and elder males (both> 0.8 and all *P* < 0.01) (Additional file 2: Figure S2a, b). In females, postmenopausal females exhibited a 40% decrease in PRL secretion compared with premenopausal women [12]. Therefore we have analyzed the performance of our model in females separately based on the menopausal status (Additional file 2: Figure S2c, d). The AUC were still higher than 0.8 in both groups (all *P* < 0.01). These data revealed that our model is efficient for identifying NAFLD regardless of aging in both genders.

To quantify the clinical contribution of PRL in the diagnosis of NAFLD, we computed two novel described metrics, IDI and NRI. The categorical NRI can determine the advancement in classification between two models by sum of the proportion of increased predicted risk in cases and the proportion of decreased predicted risk in controls. We used 0–30%, 30–60%, more than 60% to define the low-, middle-, and high-risk. The IDI can be interpreted as the difference in percentage of variance explained by the model with or without the new predictor [15, 19]. In addition, we also compared the our model with FLI in identifying hepatic steatosis, and the results showed that the efficiency of our model increased 31.95 and 26.7% in males and females, respectively (data not shown). These findings manifested that incorporation of PRL showed increased values of IDI and NRI, indicating a superiority of adding PRL within our model for predicting NAFLD.

We selected two cut-off points to improve the diagnostic accuracy, the lower cut-off point provides higher sensitivity and the higher cut-off point provides higher specificity (both higher than 90%) in diagnosing NAFLD (Table 3). This is clinically helpful because below the lower cut-off is appropriate for excluding NAFLD and subjects above the upper cut-off are more likely to present NAFLD. Although the lower cut-off (0.79 in males and 0.68 in females) provided over 90% sensitivity, the specificity in both genders were relatively low (54.4% for males and 61.3% for females). Here we would like to recommend that in subjects with risk factors for NAFLD such as elevated ALT levels or obesity, using this lower cut-off point of our model is valuable as a screening tool. On the other hand, the upper cut-off (1.71 in males and 2.16 in females) yielded higher specificity (91.1% for males and 91.4% for females) but lower sensitivity. When a clinician is about to giving a patient liver biopsy for further examination of liver lesions or treatment for NAFLD, this cut-off is useful because higher specificity can minimize the inclusion of false-positive cases. Importantly, the AUC of our model for predicting NAFLD in subjects with liver biopsy was still high: 0.71 (95%CI: 0.56–0.83) in men and 0.74 (95%CI: 0.56–0.92) in women. An AUC of more than 0.7 indicated sufficient predictive ability, of more than 0.8 indicated accurate diagnostic power [31], hence our model is optimal for the diagnosis of NAFLD in both genders. Therefore, when a patient’s score is over 1.71 in male or over 2.16 in female calculated by our model, ultrasound examination is recommended to identify these patients with high risk of developing fatty liver. However, in subjects with a score between this two cut-off points, further examination is recommended.

So far, several diagnostic algorithms have been developed to predict NAFLD, and among these the FLI has been validated in several population studies [32, 33]. FLI showed a good level of accuracy in detecting NAFLD (sensitivity of 0.84 and specificity > 0.86 for an FLI > 60) [34]. Using liver biopsy as the reference, we compared our model with FLI, and found that AUC of our model was significantly higher than that of FLI in both genders, indicating that our model was superior to FLI in terms of the predicting performance of NAFLD. Besides, the advantage of adding PRL into the model is that a positive correlation between the scores and the severity of NAFLD was observed (Fig. 2c, d), which may help the general practitioner in estimating the severe degree of NAFLD.

More importantly, since current imaging technology cannot differentiate NASH from simple steatosis, the diagnosis of NASH was still based on liver pathology [35]. It has been suggested that early liver pathology may be indicated in all NAFLD patients, since earlier intervention and more aggressive treatment can reduce overall mortality [36]. By adding the variate of PRL, our model provides an alternative method to easily identifying those with a high risk for NASH, thus may make liver biopsy unnecessary in a considerable proportion of patients.

The limitation of our study is that there is a lack of large cohort of biopsy-proven NAFLD patients, therefore our models need to be further validated in separate independent cohorts of NAFLD patients with liver pathology.

## Conclusion

In conclusion, we have established a novel non-invasive model for diagnosing the existence and severities NAFLD, which is an effective screening tool in epidemiologic studies and may help physicians identify patients for ultrasound examination.

## Supplementary information

**Additional file 1: Figure S1.** The procedure and results of screening and recruitment of the study subjects. T1DM: type 1 diabetes. n: sample sizes.

**Additional file 2: Figure S2.** (a) ROC curve in males less than 50 years old, the AUC is 0.88 (95%CI: 0.85–0.92) (*n* = 317). (b) ROC curve in males equal or greater than 50 years old, the AUC is 0.82 (95%CI: 0.75–0.89) (*n* = 135). (c) ROC curve in premenopausal females, the AUC is 0.93 (95%CI: 0.88–0.97) (*n* = 129). (d) ROC curve in postmenopausal females, the AUC is 0.79 (95%CI: 0.74–0.85) (*n* = 192).

**Additional file 3: Figure S3.** The distribution of subjects received liver biopsy. Subjects were classified into non NAFLD and NAFLD based on the proportion of affected hepatocytes. NASH was defined if NAS score was ≥5, and excluded if NAS score was less than 3. NASH: nonalcoholic steatohepatitis.

**Additional file 4: Table S1.** Baseline characteristics of all the study population.

**Additional file 5: Table S2.** Demographic and biochemical features of premenopausal and postmenopausal females.

**Additional file 6: Table S3.** Clinical and laboratory data of subjects with liver biopsy.

**Additional file 7: Table S4.** Clinical and laboratory data of participants with and without NASH.

**Additional file 8: Table S5.** Comparison of diagnostic performance of the formula with and without PRL.

## Data Availability

Data and materials are not available in this study for the protection of subjects’ privacy, and are available from the corresponding author on reasonable requests.

## Abbreviations

ALT: Aminotransferas
AST: Aspartate aminotransferase
AUC: Area under receiver operating characteristic
BMI: Body mass index
CI: Confidential interval
FBG: Fasting plasma glucose
FLI: Fatty liver index
GCT: γ-glutamyl-transferase
HD: High-density lipoprotein
ID: Integrated discrimination improvement
IQR: Interquartile range
LDL: Low-density lipoprotein
NAFLD: Non-alcoholic fatty liver disease
NPV: Negative predictive values
NRI: Categorical net reclassification improvement
PP: Positive predictive values
PRL: Prolactin
PRLR: Receptor
ROC: Receiver operating characteristic
STARD: Standards for reporting diagnostic accuracy
TC: Total cholesterol
TG: Triglycerides
WC: Waist circumference
