# Correction to: A newly noninvasive model for prediction of non-alcoholic fatty liver disease: utility of serum prolactin levels

**DOI:** 10.1186/s12876-020-1181-z

**Published:** 2020-02-18

**Authors:** Pengzi Zhang, Wenhuan Feng, Xuehui Chu, Xitai Sun, Dalong Zhu, Yan Bi

**Affiliations:** 1 Department of Endocrinology, Drum Tower Hospital Affiliated to Nanjing University Medical School, No 321, Zhongshan Road, Nanjing, 210008 Jiangsu China; 2 Department of General Surgery, Drum Tower Hospital Affiliated to Nanjing University Medical School, Nanjing, China

**Correction to: BMC Gastroenterol**

**https://doi.org/10.1186/s12876-019-1120-z**

Following publication of the original article [1], we have been notified that the given name of one of the authors was spelled incorrectly. It is now Wenghuan Feng and should be as follows:

Wenhuan Feng

The original article has been corrected.
