# Supplementary figures and images for: A newly noninvasive model for prediction of non-alcoholic fatty liver disease: utility of serum prolactin levels
Source: BMC Gastroenterol. 2019 Nov 27;19:202. doi: 10.1186/s12876-019-1120-z (PMC6882057; doi:10.1186/s12876-019-1120-z)

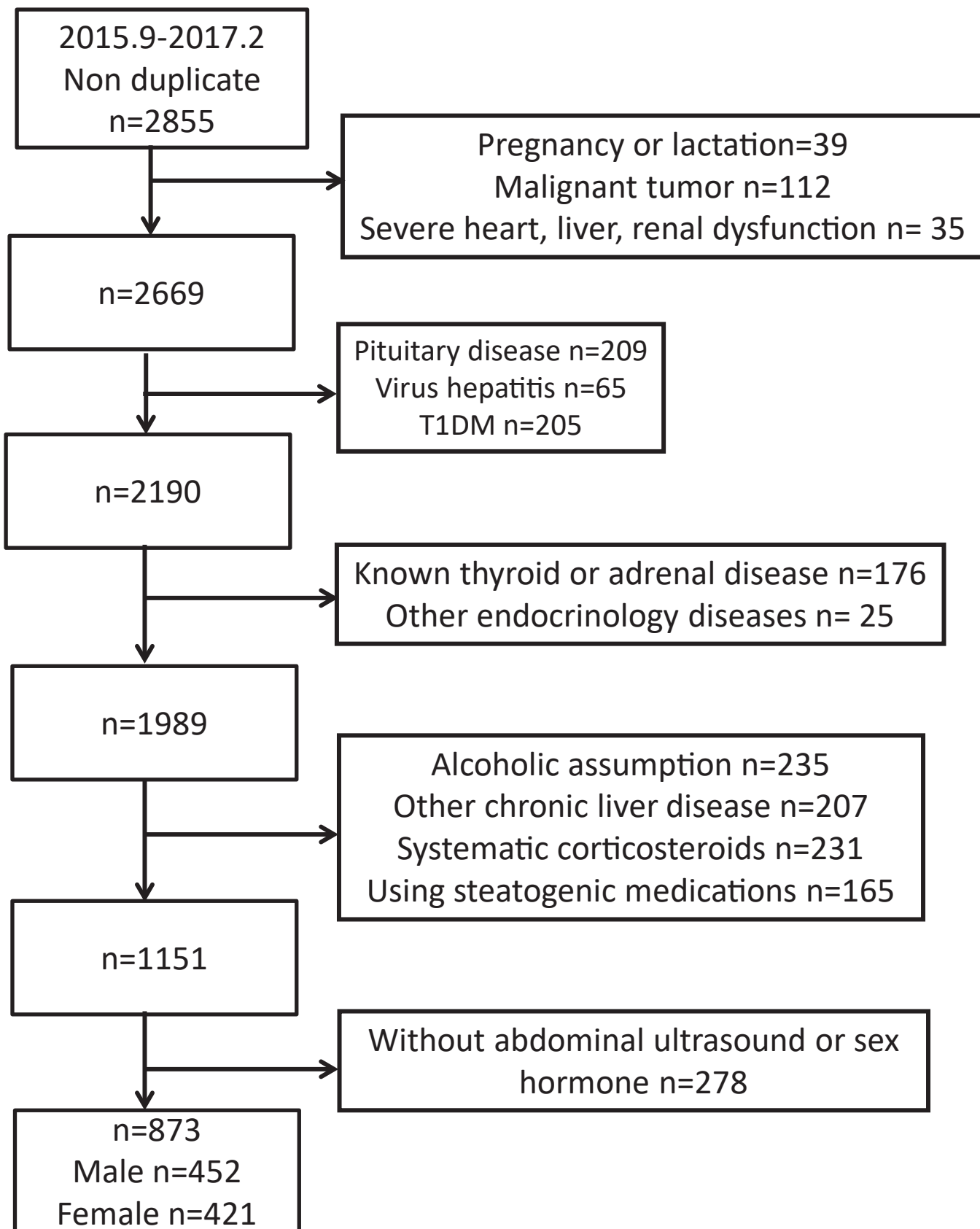

Supplement: Supplementary file 1 — Additional file 1: Figure S1. The procedure and results of screening and recruitment of the study subjects. T1DM: type 1 diabetes. n: sample sizes. [file 12876_2019_1120_MOESM1_ESM.pdf]

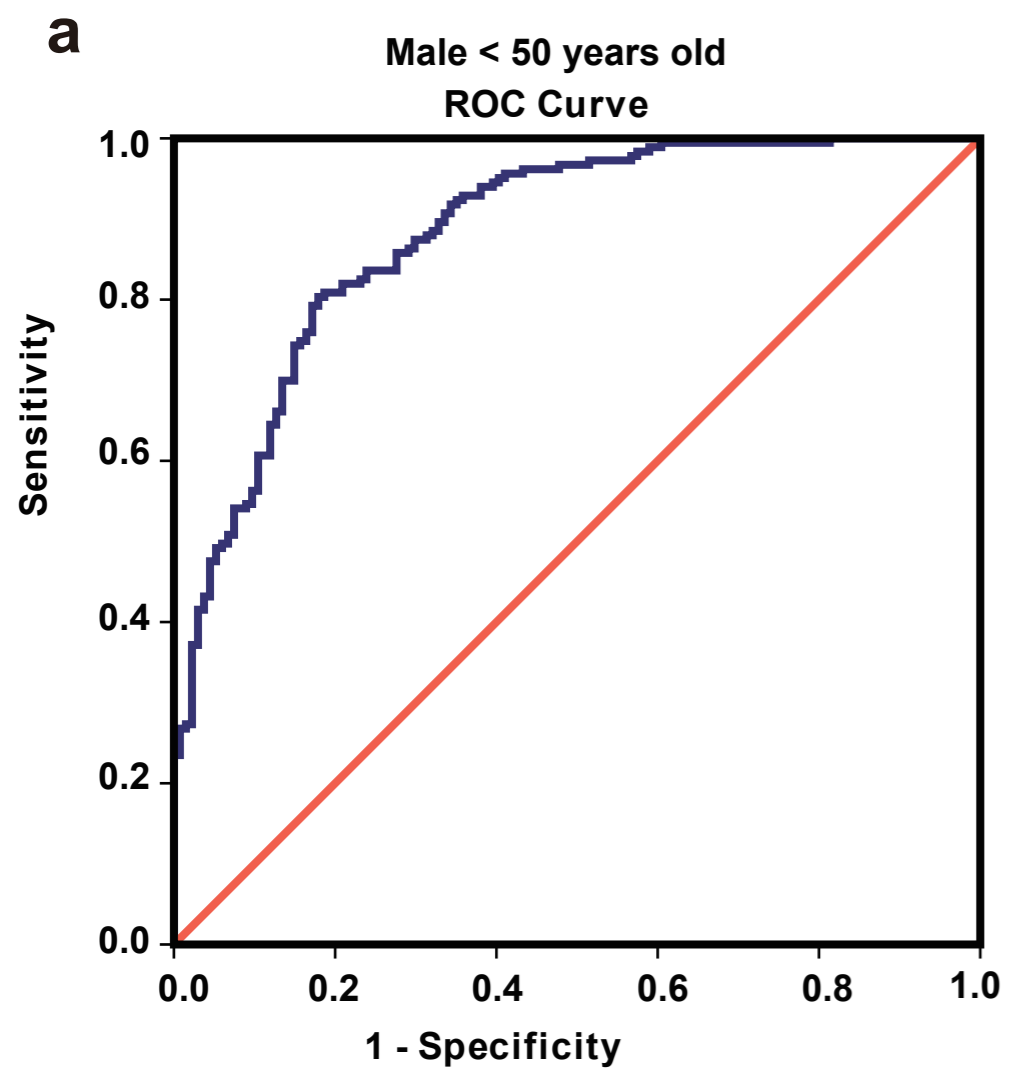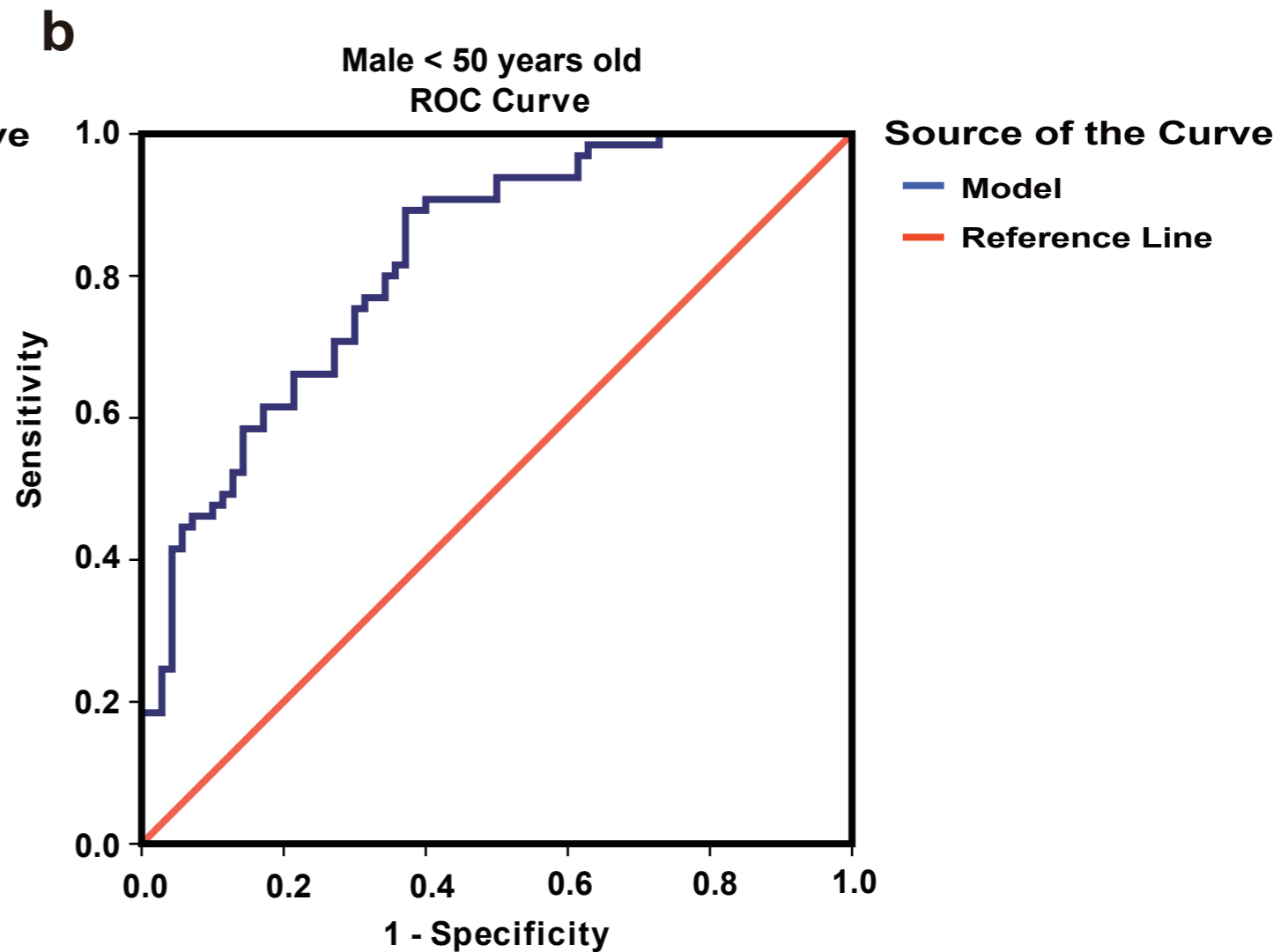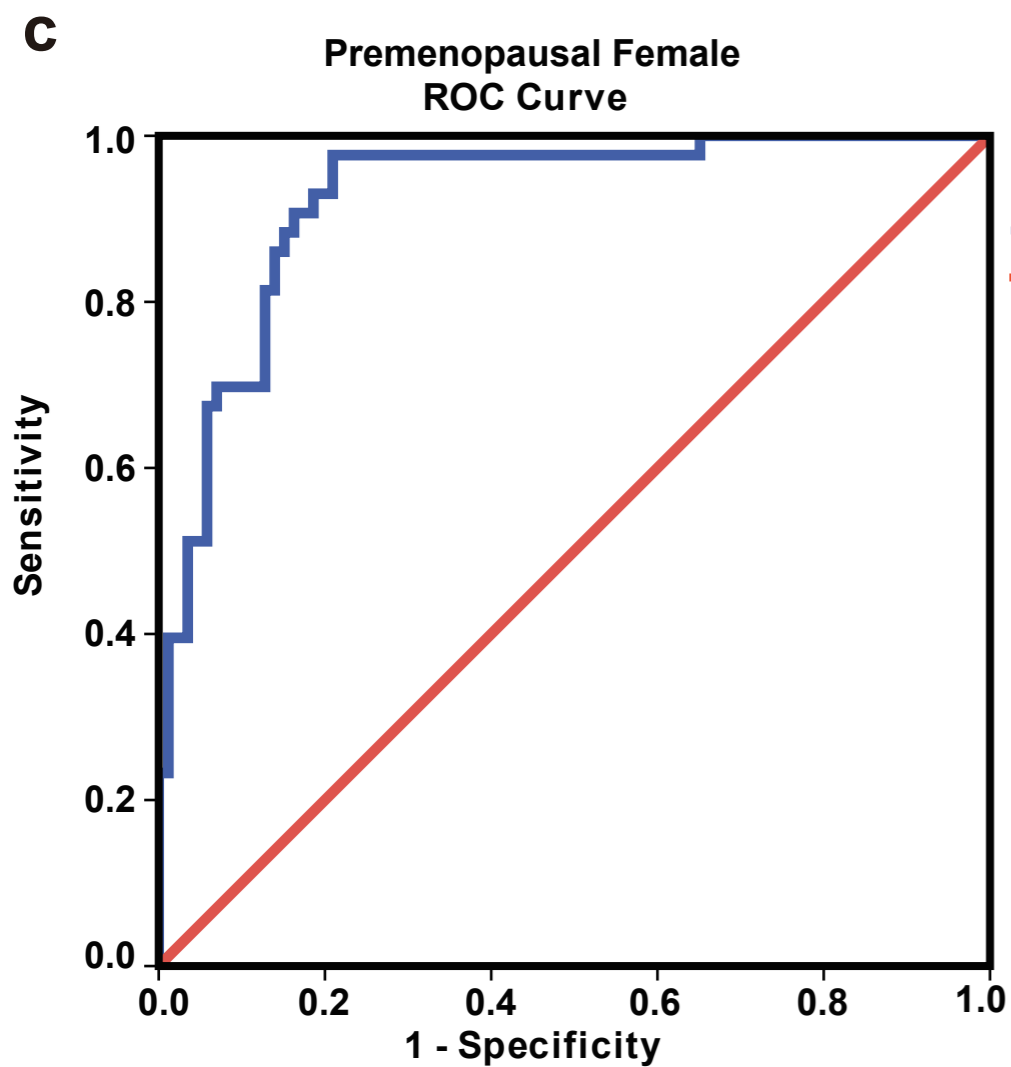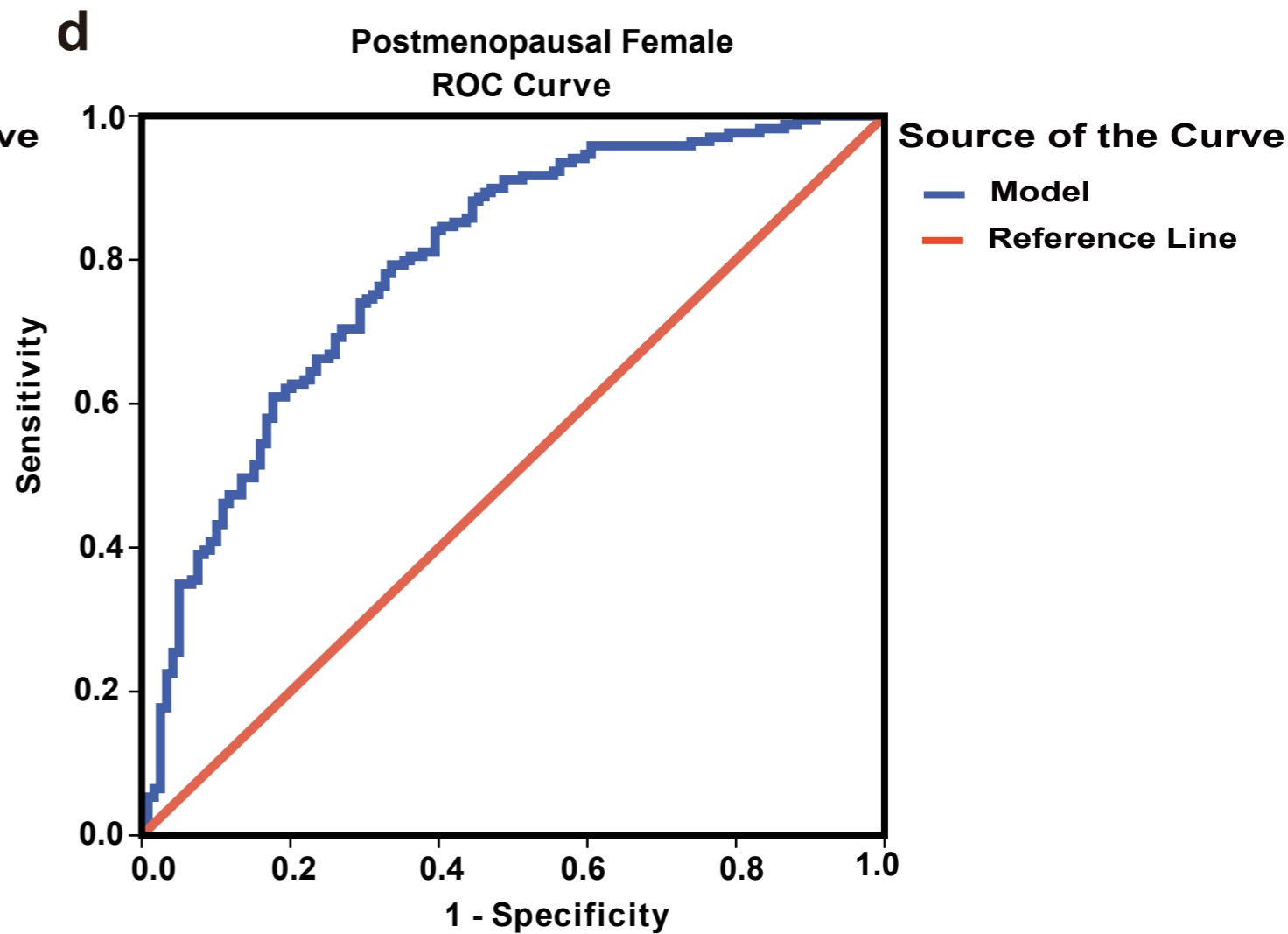

Supplement: Supplementary file 2 — Additional file 2: Figure S2. (a) ROC curve in males less than 50 years old, the AUC is 0.88 (95%CI: 0.85–0.92) (n = 317). (b) ROC curve in males equal or greater than 50 years old, the AUC is 0.82 (95%CI: 0.75–0.89) (n = 135). (c) ROC curve in premenopausal females, the AUC is 0.93 (95%CI: 0.88–0.97) (n = 129). (d) ROC curve in postmenopausal females, the AUC is 0.79 (95%CI: 0.74–0.85) (n = 192). [file 12876_2019_1120_MOESM2_ESM.pdf]
