# Supplementary material for: A newly noninvasive model for prediction of non-alcoholic fatty liver disease: utility of serum prolactin levels
Source: BMC Gastroenterol. 2019 Nov 27;19:202. doi: 10.1186/s12876-019-1120-z (PMC6882057; doi:10.1186/s12876-019-1120-z)
Supplement: Supplementary file 3 — Additional file 3: Figure S3. The distribution of subjects received liver biopsy. Subjects were classified into non NAFLD and NAFLD based on the proportion of affected hepatocytes. NASH was defined if NAS score was ≥5, and excluded if NAS score was less than 3. NASH: nonalcoholic steatohepatitis. [file 12876_2019_1120_MOESM3_ESM.pdf]

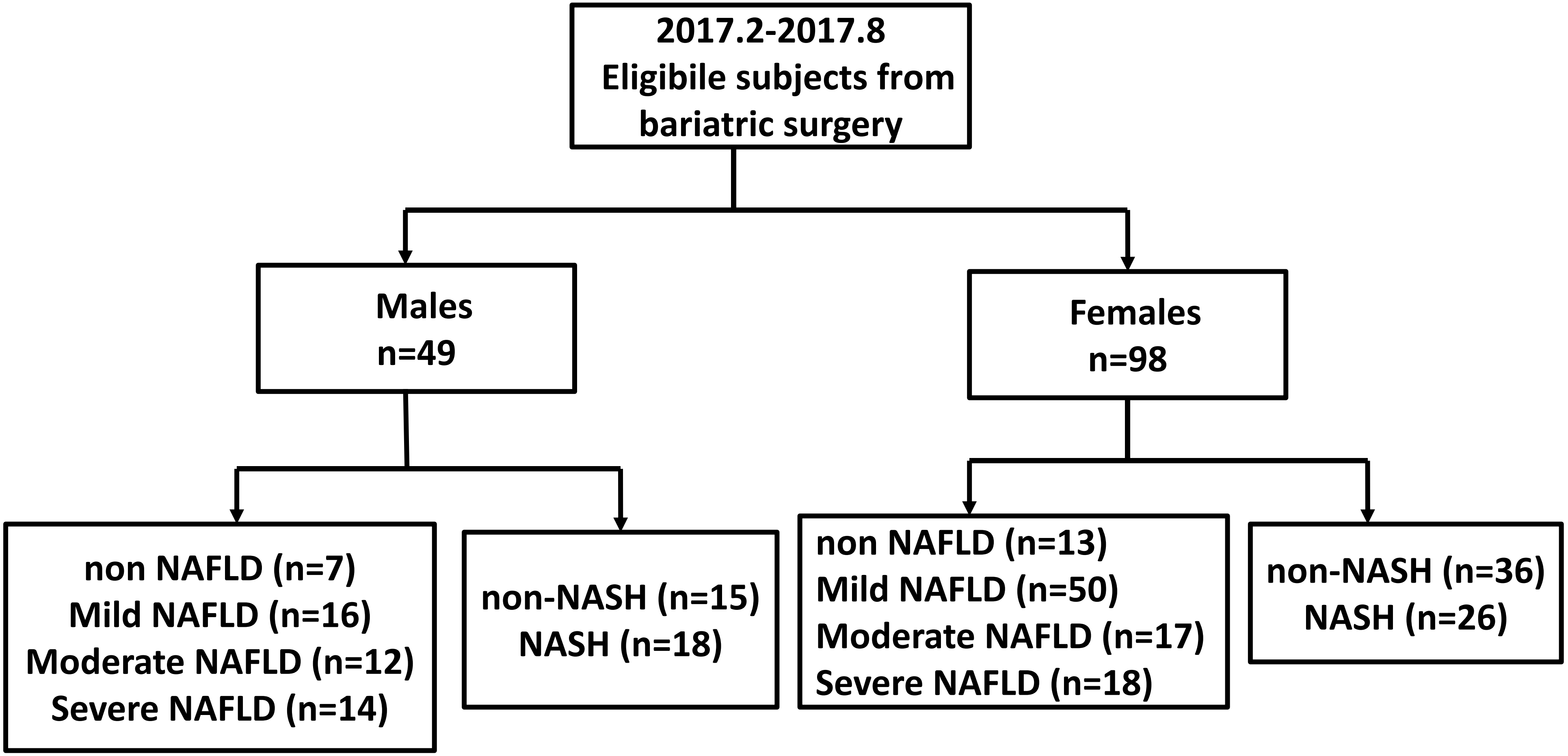
2017.2-2017.8
Eligibile subjects from bariatric surgery
Males
n=49
Females
n=98
non NAFLD (n=7)
Mild NAFLD (n=16)
Moderate NAFLD (n=12)
Severe NAFLD (n=14)
non-NASH (n=15)
NASH (n=18)
non NAFLD (n=13)
Mild NAFLD (n=50)
Moderate NAFLD (n=17)
Severe NAFLD (n=18)
non-NASH (n=36)
NASH (n=26)
