# Supplementary material for: A newly noninvasive model for prediction of non-alcoholic fatty liver disease: utility of serum prolactin levels
Source: BMC Gastroenterol. 2019 Nov 27;19:202. doi: 10.1186/s12876-019-1120-z (PMC6882057; doi:10.1186/s12876-019-1120-z)
Supplement: Supplementary file 4 — Additional file 4: Table S1. Baseline characteristics of all the study population. [file 12876_2019_1120_MOESM4_ESM.doc]

**Table S1 Baseline characteristics of all the study population**

|  | **Men** | |  | **Women** | |  |
| --- | --- | --- | --- | --- | --- | --- |
|  | **Non-NAFLD** | **NAFLD** | ***P*** | **Non-NAFLD** | **NAFLD** | ***P*** |
| **N** | 204 | 248 |  | 205 | 216 |  |
| **Age (years)** | 55 (47, 65) | 52 (41, 60.8) | 0.08 | 57 (45.5, 65) | 60 (52, 68) | <0.01 |
| **BMI (kg/m^2^)** | 23.4 (21.8, 25.1) | 26.4 (24.8, 28.1) | <0.01 | 23.4 (21.2, 25.3) | 26 (24.1, 29) | <0.01 |
| **SBP (mmHg)** | 132 (119, 144) | 136 (125, 147) | 0.01 | 129 (119.5, 143.5) | 139 (128.5, 151) | <0.01 |
| **DBP (mmHg)** | 78 (71, 87) | 84 (77, 91) | <0.01 | 76 (67, 85) | 81 (73.5, 91) | 0.23 |
| **Waist (cm)** | 89 (84, 95) | 97 (92, 100) | <0.01 | 87 (80, 91) | 94 (87, 99.5) | <0.01 |
| **HbA1c (%)** | 7.3 (5.9, 10.1) | 7.6 (6.5, 9.4) | <0.01 | 7.3 (5.9, 8.5) | 8.2 (6.8, 9.8) | <0.01 |
| **FBG (mmol/L)** | 6.4 (5, 8.3) | 7.5 (5.8, 9.5) | <0.01 | 6.3 (5.1, 7) | 7.6 (6.2, 9.7) | <0.01 |
| **ALT (U/L)** | 19.2 (13.5, 26.7) | 28.1 (19.8, 43.8) | <0.01 | 14.7 (11.5, 19.2) | 23.7 (17, 35.8) | <0.01 |
| **AST (U/L)** | 17.1 (14.8, 21.3) | 20.3 (16.4, 27.8) | <0.01 | 16.3 (13.3, 20.9) | 20.4 (17, 26.5) | <0.01 |
| **TG (mmol/L)** | 1.2 (0.9, 1.5) | 1.9 (1.3, 2.8) | <0.01 | 1.2 (0.9, 1.9) | 1.7 (1.3, 2.4) | <0.01 |
| **TC (mmol/L)** | 4.3 (3.5, 4.9) | 4.5 (3.8, 5.1) | 0.01 | 4.5 (3.6, 5.3) | 4.6 (3.9, 5.2) | 0.02 |
| **HDL (mmol/l)** | 1.1 (0.9, 1.3) | 0.9 (0.8, 1.1) | <0.01 | 1.2 (1.0, 1.445) | 1.1 (1.0, 1.2) | <0.01 |
| **LDL (mmol/l)** | 2.4 (1.8, 3) | 2.4 (1.9, 3) | 0.20 | 2.6 (1.9, 3.2) | 2.7 (2.0, 3.1) | 0.01 |
| **PRL (ug/L)** | 9 (7.1, 13.4) | 7.8 (5.9, 10.4) | <0.01 | 10.5 (8, 14.9) | 8.6 (6, 11.2) | <0.01 |

BMI: body mass index; SBP: systolic blood pressure; DBP: diastolic blood pressure; FBG: fasting blood glucose; HbA1c: haemoglobin 1c; ALT: alanine aminotransferase; AST: aspartate transaminase (AST); HDL: high-density lipoprotein; LDL: low-density lipoprotein; NAFLD: non-alcoholic fatty liver disease; PRL: prolactin; TC: total cholesterol; TG: triglyceride; Data are shown as median with interquartile range (IQR). *p* values are based on Mann-Whitney U test
