# Supplementary material for: A newly noninvasive model for prediction of non-alcoholic fatty liver disease: utility of serum prolactin levels
Source: BMC Gastroenterol. 2019 Nov 27;19:202. doi: 10.1186/s12876-019-1120-z (PMC6882057; doi:10.1186/s12876-019-1120-z)
Supplement: Supplementary file 5 — Additional file 5: Table S2. Demographic and biochemical features of premenopausal and postmenopausal females. [file 12876_2019_1120_MOESM5_ESM.doc]

**Table S2 Demographic and biochemical features of premenopausal and postmenopausal females**

|  | **Men** | |  | **Women** | |  |
| --- | --- | --- | --- | --- | --- | --- |
|  | **<50 years** | **≥50 years** | ***P*** | **Premenopausal** | **Postmenopausal** | ***P*** |
| **N** | 317 | 135 |  | 129 | 292 |  |
| **Age (years)** | 47 (38, 53) | 65 (62, 70) | <0.01 | 35 (27, 44) | 63 (56, 70) | <0.01 |
| **BMI (kg/m^2^)** | 25.6 (23.4, 27.4) | 24.9 (23.1, 27.3) | 0.12 | 134 (123, 148) | 136.5 (126, 152) | <0.01 |
| **SBP (mmHg)** | 131 (119, 144) | 141 (129.3, 150.8) | <0.01 | 88 (76, 96.5) | 78 (70, 86) | <0.01 |
| **DBP (mmHg)** | 83 (75, 92) | 79 (72.3, 87) | 0.01 | 25 (21.5, 28) | 24.6 (22.3, 27.4) | 0.59 |
| **Waist (cm)** | 94 (88, 98) | 95 (88.3, 99) | 0.46 | 86 (77, 94) | 90 (83, 98) | 0.06 |
| **HbA1c (%)** | 7.3 (6, 9.5) | 8.1 (6.6, 10) | <0.01 | 4.9 (4.5, 6.4) | 6.9 (5.3, 9.1) | <0.01 |
| **FBG (mmol/L)** | 7 (5.4, 9.1) | 7.1 (5.6, 9.1) | 0.16 | 6 (5, 7.5) | 7.9 (6.4, 9.4) | <0.01 |
| **ALT (U/L)** | 26.7 (17.4, 42.2) | 20.4 (15.4, 28.6) | <0.01 | 20 (13, 36) | 19.9 (14.5, 28.7) | 0.49 |
| **AST (U/L)** | 19.1 (15.5, 26.1) | 18.2 (15.6, 22.4) | 0.22 | 19 (15, 25.5) | 18.5 (15, 24.1) | 0.55 |
| **TG (mmol/L)** | 1.7 (1.2, 2.5) | 1.3 (1, 1.9) | <0.01 | 1.4 (0.9, 1.9) | 1.5 (1.2, 2.2) | <0.01 |
| **TC (mmol/L)** | 4.5 (3.8, 5.2) | 4.3 (3.4, 4.9) | 0.10 | 4.3 (3.8, 4.9) | 4.6 (4, 5.3) | <0.01 |
| **HDL (mmol/l)** | 0.96 (0.8, 1.1) | 1.02 (0.8, 1.3) | 0.01 | 1.1 (1, 1.4) | 1.1 (1, 1.4) | 0.88 |
| **LDL (mmol/l)** | 2.5 (1.9, 3) | 2.3 (1.8, 2.9) | 0.13 | 2 (2, 3) | 2.7 (2.1, 3.2) | <0.01 |
| **PRL (ug/L)** | 8.5 (6.5, 12) | 8 (6.1, 10.3) | 0.13 | 13 (10, 16) | 8.6 (6.3, 10.9) | <0.01 |

BMI: body mass index; SBP: systolic blood pressure; DBP: diastolic blood pressure; FBG: fasting blood glucose; HbA1c: haemoglobin 1c; ALT: alanine aminotransferase; AST: aspartate transaminase (AST); HDL: high-density lipoprotein; LDL: low-density lipoprotein; NAFLD: non-alcoholic fatty liver disease; PRL: prolactin; TC: total cholesterol; TG: triglyceride. Data are shown as median with interquartile range (IQR). *p* values are based on Mann-Whitney U test
