# Supplementary material for: A newly noninvasive model for prediction of non-alcoholic fatty liver disease: utility of serum prolactin levels
Source: BMC Gastroenterol. 2019 Nov 27;19:202. doi: 10.1186/s12876-019-1120-z (PMC6882057; doi:10.1186/s12876-019-1120-z)
Supplement: Supplementary file 6 — Additional file 6: Table S3. Clinical and laboratory data of subjects with liver biopsy. [file 12876_2019_1120_MOESM6_ESM.doc]

**Table S3 Clinical and laboratory data of subjects with liver biopsy**

|  | **Men** | |  | **Women** | |  |
| --- | --- | --- | --- | --- | --- | --- |
|  | **Non-NAFLD** | **NAFLD** | ***P*** | **Non-NAFLD** | **NAFLD** | ***P*** |
| **N** | 7 | 42 |  | 13 | 85 |  |
| **Age (years)** | 37 (28, 46.8) | 32 (28, 41) | 0.38 | 31.5 (21.5, 42.5) | 33.5 (29, 48) | 0.16 |
| **BMI (kg/m^2^)** | 42.1 (32.6, 48.5) | 40 (36.2, 44.5) | 0.50 | 124 (112.5, 143.3) | 132.5 (123.3, 142) | 0.11 |
| **SBP (mmHg)** | 168.5 (158, 180.5) | 143.5 (132.5, 158) | 0.43 | 77.5 (71.8, 91.5) | 82 (73, 91) | 0.36 |
| **DBP (mmHg)** | 104.5 (97.5, 120.5) | 88 (81.3, 100.8) | 0.18 | 33.9 (32.2, 45) | 35.9 (32.5, 40.5) | 0.27 |
| **Waist (cm)** | 124.5 (110.3, 140.3) | 122 (114, 129.8) | 0.89 | 107.5 (91.5, 127.8) | 110 (103.3, 124.3) | 0.39 |
| **HbA1c (%)** | 6.2 (5.1, 8.3) | 6.5 (5.8, 8.3) | 0.08 | 5.3 (4.7, 5.9) | 5.6 (5.1, 7.5) | 0.047 |
| **FBG (mmol/L)** | 5.5 (4.5, 8.3) | 6.7 (5.3, 9.1) | 0.03 | 5.1 (4.8, 5.7) | 5.6 (5.2, 7.6) | 0.04 |
| **ALT (U/L)** | 22.1 (15.4, 29.6) | 55.5 (37.2, 99.7) | 0.01 | 23.9 (14.2, 30.1) | 35.4 (22.5, 60.3) | 0.01 |
| **AST (U/L)** | 20.8 (14.1, 26.8) | 30.5 (22.3, 44.3) | 0.02 | 21.3 (15.5, 25.6) | 23.7 (17.6, 38.6) | 0.15 |
| **TG (mmol/L)** | 2 (1.5, 3.3) | 2.2 (1.6, 2.8) | 0.25 | 1.3 (1, 1.8) | 1.6 (1.1, 2.3) | 0.07 |
| **TC (mmol/L)** | 5.4 (4.7, 5.8) | 4.7 (4.2, 5.6) | 0.67 | 4.6 (3.6, 5.1) | 4.7 (4.1, 5.3) | 0.26 |
| **HDL (mmol/l)** | 1 (0.8, 1.3) | 0.9 (0.8, 1) | 0.98 | 1 (0.9, 1.5) | 1 (0.9, 1.3) | 0.84 |
| **LDL (mmol/l)** | 3.2 (2.7, 3.4) | 2.9 (2.3, 3.3) | 0.36 | 2.4 (1.8, 3.2) | 2.8 (2.2, 3.2) | 0.28 |
| **PRL (ug/L)** | 8.3 (5.8, 18.3) | 8.5 (6.3, 10.4) | 0.53 | 16.9 (10.8, 21) | 9.8 (6.1, 14.8) | <0.01 |
| **NAS score** | 1.0 (1.0, 2.5) | 4.0 (3.0, 5.0) | <0.01 | 2.0 (1.0, 2.0) | 3.0 (2.0, 5.0) | <0.01 |

BMI: body mass index; SBP: systolic blood pressure; DBP: diastolic blood pressure; FBG: fasting blood glucose; HbA1c: haemoglobin 1c; ALT: alanine aminotransferase; AST: aspartate transaminase (AST); HDL: high-density lipoprotein; LDL: low-density lipoprotein; NAFLD: non-alcoholic fatty liver disease; PRL: prolactin; TC: total cholesterol; TG: triglyceride; NAS: NAFLD activity score. Data are shown as median with interquartile range (IQR). NAS: NAFLD activity score. *p* values are based on Mann-Whitney U test.
