# Supplementary material for: A newly noninvasive model for prediction of non-alcoholic fatty liver disease: utility of serum prolactin levels
Source: BMC Gastroenterol. 2019 Nov 27;19:202. doi: 10.1186/s12876-019-1120-z (PMC6882057; doi:10.1186/s12876-019-1120-z)
Supplement: Supplementary file 7 — Additional file 7: Table S4. Clinical and laboratory data of participants with and without NASH. [file 12876_2019_1120_MOESM7_ESM.doc]

**Table S4 Clinical and laboratory data of participants with and without NASH.**

|  | **Men** | |  | **Women** | |  |
| --- | --- | --- | --- | --- | --- | --- |
|  | **Non-NASH** | **NASH** | ***P*** | **Non-NASH** | **NASH** | ***P*** |
| **N** | 15 | 18 |  | 36 | 26 |  |
| **Age (years)** | 41 (28, 46) | 29.5 (26.8, 36) | 0.29 | 32 (29.8, 43) | 30.5 (26, 38) | 0.25 |
| **BMI (kg/m^2^)** | 34.9 (31.5, 40.7) | 42.5 (39, 48.8) | <0.01 | 35.8 (32.6, 45.8) | 38.1 (35.3, 41.4) | 0.25 |
| **SBP (mmHg)** | 149 (130, 164) | 141.5 (131.3, 159.8) | 0.69 | 131 (120.8, 138.8) | 138.5 (127.3, 143) | 0.04 |
| **DBP (mmHg)** | 90 (87, 107) | 87.5 (79.8, 101.8) | 0.79 | 79.5 (73, 86) | 84 (75.5, 92.3) | 0.33 |
| **Waist (cm)** | 114 (107, 123) | 127.5 (114.8, 145.3) | 0.02 | 109 (106, 128) | 117 (107.3, 132.3) | 0.30 |
| **HbA1c (%)** | 6.9 (6.1, 8.8) | 6.3 (5.8, 8.2) | 0.70 | 6 (5, 6.3) | 6.7 (6, 8) | 0.00 |
| **FBG (mmol/L)** | 8.1 (5.3, 10.8) | 7 (5.1, 8.4) | 0.97 | 5.3 (4.8, 6.1) | 6.2 (5.3, 8.9) | 0.01 |
| **ALT (U/L)** | 25.7 (23.3, 51.9) | 90.5 (47.5, 149.1) | <0.01 | 25.4 (20, 39.7) | 67.8 (30.6, 104.9) | <0.01 |
| **AST (U/L)** | 19.7 (15.7, 24) | 36.5 (29.2, 65.8) | <0.01 | 22 (16.2, 26.3) | 39.1 (21.6, 71.7) | <0.01 |
| **TG (mmol/L)** | 2.1 (1.6, 2.5) | 2.4 (1.6, 3.4) | 0.29 | 1.4 (1.1, 1.8) | 1.9 (1.6, 2.4) | 0.01 |
| **TC (mmol/L)** | 5.1 (4.4, 5.4) | 5.2 (4.5, 6.6) | 0.26 | 4.7 (3.7, 5.3) | 4.8 (4.2, 5.5) | 0.57 |
| **HDL (mmol/l)** | 1 (0.8, 1.2) | 0.9 (0.7, 1) | 0.29 | 1 (0.9, 1.2) | 1 (0.8, 1.2) | 0.28 |
| **LDL (mmol/l)** | 2.9 (2.5, 3.3) | 3.1 (2.2, 3.5) | 0.75 | 3 (2.2, 3.3) | 2.9 (2.3, 3.4) | 0.95 |
| **PRL (ug/L)** | 8.3 (5.4, 12.6) | 8.7 (6.1, 9.7) | 0.97 | 12 (8.8, 17.4) | 10.9 (7.5, 13.7) | 0.34 |
| **NAS score** | 1.5 (1.0, 2.3) | 4.0 (3.0, 5.0) | <0.01 | 2.0 (1.0, 2.0) | 5.0 5.0, 6.0) | <0.01 |

BMI: body mass index; SBP: systolic blood pressure; DBP: diastolic blood pressure; FBG: fasting blood glucose; HbA1c: haemoglobin 1c; ALT: alanine aminotransferase; AST: aspartate transaminase (AST); HDL: high-density lipoprotein; LDL: low-density lipoprotein; NAFLD: non-alcoholic fatty liver disease; PRL: prolactin; TC: total cholesterol; TG: triglyceride; NAS: NAFLD activity score. Data are shown as median with interquartile range (IQR). NAS: NAFLD activity score. *p* values are based on Mann-Whitney U test.
