# Supplementary material for: A newly noninvasive model for prediction of non-alcoholic fatty liver disease: utility of serum prolactin levels
Source: BMC Gastroenterol. 2019 Nov 27;19:202. doi: 10.1186/s12876-019-1120-z (PMC6882057; doi:10.1186/s12876-019-1120-z)
Supplement: Supplementary file 8 — Additional file 8: Table S5. Comparison of diagnostic performance of the formula with and without PRL. [file 12876_2019_1120_MOESM8_ESM.doc]

**Table S5 Comparison of diagnostic performance of the formula with and without PRL**

| **Male** | **Formula** | 0.469*BMI (kg/m^2^) + 0.028*ALT (U/l) -2.236*HDL (mmol/l)-9.838 | |
| --- | --- | --- | --- |
|  | **IDI** | Value | 0.196 |
|  |  | 95%CI | 0.052-0.340 |
|  |  | *P* | 0.008 |
|  | **NRI** | Value | 0.119 |
|  |  | 95%CI | -0.361-0.599 |
|  |  | *P* | 0.627 |
| **Female** | **Formula** | 0.264*BMI (kg/m^2^) + 0.072*waist (cm) + 0.481*HbA1c (%) +0.084*ALT (U/l)-17.467 | |
|  | **IDI** | Value | 0.262 |
|  |  | 95%CI | 0.090-0.434 |
|  |  | *P* | 0.003 |
|  | **NRI** | Value | 0.500 |
|  |  | 95%CI | 0.187-0.813 |
|  |  | *P* | 0.002 |

BMI: body mass index; HbA1c: haemoglobin 1c; ALT: alanine aminotransferase; AST: aspartate transaminase (AST); HDL: high-density lipoprotein; IDI: the integrated discrimination improvement; NRI: categorical net reclassification improvement;
